# High Power Impulse Magnetron Sputtering of In_2_O_3_/Sn Cold Sprayed Composite Target

**DOI:** 10.3390/ma14051228

**Published:** 2021-03-05

**Authors:** Marcin Winnicki, Artur Wiatrowski, Michał Mazur

**Affiliations:** 1 Department of Metal Forming, Welding and Metrology, Wrocław University of Science and Technology, Lukasiewicza 5, 50-371 Wroclaw, Poland; 2 Department of Microelectronics and Nanotechnology, Wrocław University of Science and Technology, Janiszewskiego 11/17, 50-372 Wroclaw, Poland; artur.wiatrowski@pwr.edu.pl (A.W.); michal.mazur@pwr.edu.pl (M.M.)

**Keywords:** HiPIMS technique, hybrid-type metal–ceramic target, ITO, cold spray, composite coating

## Abstract

High Power Impulse Magnetron Sputtering (HiPIMS) was used for deposition of indium tin oxide (ITO) transparent thin films at low substrate temperature. A hybrid-type composite target was self-prepared by low-pressure cold spraying process. Prior to spraying In_2_O_3_ and oxidized Sn powders were mixed in a volume ratio of 3:1. Composite In_2_O_3_/Sn coating had a mean thickness of 900 µm. HiPIMS process was performed in various mixtures of Ar:O_2_: (i) 100:0 vol.%, (ii) 90:10 vol.%, (iii) 75:25 vol.%, (iv) 50:50 vol.%, and (v) 0:100 vol.%. Oxygen rich atmosphere was necessary to oxidize tin atoms. Self-design, simple high voltage power switch capable of charging the 20 µF capacitor bank from external high voltage power supply worked as a power supply for an unbalanced magnetron source. ITO thin films with thickness in the range of 30–40 nm were obtained after 300 deposition pulses of 900 V and deposition time of 900 s. The highest transmission of 88% at λ = 550 nm provided 0:100 vol. % Ar:O_2_ mixture, together with the lowest resistivity of 0.03 Ω·cm.

## 1. Introduction

In recent years, a development of nanomaterial-based semiconductor caused dynamic progress in fabrication of more advanced electronic systems [1,2,3]. Indium-tin oxide (ITO), a heavily doped and highly degenerated n-type semiconductor with high carrier concentration (~10^21^ cm^−3^) [4,5,6], is one of the most widely used transparent conductive oxides (TCO) due to unique combination of excellent electrical conductivity, optical transparency and good mechanical properties and relatively good chemical stability [7,8,9]. Although various new materials, such as tin dioxide (SnO_2_) [10], zinc oxide (ZnO) [11,12], indium zinc oxide (IZO) [13], conductive nano-silver wire [14], have been applied in industry, ITO is still the main choice for conductive optical [15]. Its excellent photoelectric performance demonstrate potential in high efficiency optoelectronic devices including solar cells [16], touch screens [17], panel displays [17], organic light emitting diodes [18], electro-optic switches [19], liquid crystal devices (LCDs) [20], but also sensors for electronic skins [12] or thin film photovoltaics [21].

Technologies of unmodified ITO film deposition on glass panels are well developed and comprise physical vapour deposition (PVD) [22], chemical vapor deposition (CVD) [23], spin-coating [24], spray pyrolysis [25] and ultrasonic spray [26]. Nevertheless, liquid techniques, such as spin-coating, spray pyrolysis or ultrasonic spray, require additional post-deposition annealing process to sinter nanoparticles, in order to achieve the specific properties of material, particularly low electrical resistance [27,28]. Additional furnace heating elongates the ITO fabrication time and increases the costs as well. To shorten the time other heat-treatment (HT) processes can be used, e.g., laser annealing proposed by Park and Kim [29] to sinter spin-coated ITO nanoparticles (NP). Unfortunately, achieving low electrical resistivity required only tens of seconds and as a result the control of HT was difficult. Therefore, a still dominating processes of ITO films deposition are PVD, e.g., magnetron sputtering (MS) [22] and vacuum evaporation [30], or CVD [23]. Target, a feedstock material used in PVD processes, is ceramic (In_2_O_3_-SnO_2_ sinter) or metallic (In-Sn alloy) [31]. The former material provides higher performance, while the latter one is sputtered with Ar-O_2_ mixture and requires additional control. Nevertheless, ceramic targets exhibit some imperfections, such as: (i) non-uniformity of chemical composition across the target body that favour formation of black deposit, known as a nodule, which destabilize sputtering process and affect final properties of the deposited film [32,33], (ii) possible wrapping or cracking due to powders hot pressing in production process [34,35], and (iii) induction of the particle arcing events resulting from intensely focused and localized discharge of particles, created nodules, flakes, or impurities on target surface [33,35]. It should be noted that commercially available targets consist of two soldered elements, such as blank and backing plate. When the bonding is unstable, the target cracks in the sputtering process and causes contamination of the film [36]. Described above problems can be eliminated by preparation of a new type of target or modification of deposition process.

A high potential in targets manufacturing revealed cold spraying [37], a promising additive manufacturing technology dedicated for metal and metal–ceramic parts fabrication [38]. Cold spraying is a solid-state process used for the deposition of dense and uniform layers via powder plastic deformation. Preheated gas stream accelerates powder particles in de Laval nozzle to supersonic velocity. Impacting with high kinetic energy particles deform and mechanically interlock on substrate surface [39]. Based on the process nature, admixture of ceramic to metal powder decreases the porosity in the layers [40]. It is worth stressing, that high density and uniformity is favourable for ITO targets. Furthermore, combining a high-quality target with appropriate process can further improve properties of deposited films. A perspective approach concerns ionized sputtering achievable in high power impulse magnetron sputtering (HiPIMS).

HiPIMS is a plasma-based modern physical vapor deposition (PVD) technique that uses ions instead of neutrals, or at least a significant fraction of ionized species, for thin film deposition. This relatively new technology is characterized by very high peak power density (>1 kW/cm^2^) at the sputtering target and high plasma density (>10^19^ m^3^) in front of the target with very short variable pulse durations (in the range of 50–200 μs) [41]. It results in high degree of ionization (up to 90%) and high ion-flux towards the substrate with simultaneous lower process temperature. Consequently, HiPIMS has widened the potential application areas of MS for deposition of high-quality thin films with denser structure, smoother grain size, and higher mechanical properties compared to conventional MS [42].

In this work, we applied HiPIMS to deposit ITO films on glass substrates with the use of self-prepared Sn+In_2_O_3_ targets. Our previous study [37] showed that fully transparent ITO films with the thickness up to 200 nm and resistance of 1 MΩ (sheet resistance of 1 × 10^5^ Ω/sq) can be deposited by conventional magnetron sputtering using targets produced by cold spraying (CS). According to the bibliography research, there are no papers describing the implementation of HiPIMS process in the deposition of ITO films with a hybrid-type metal–ceramic targets. Therefore, the main attention is focused on the concentration of oxygen in the Ar:O_2_ mixture, which oxidizes tin and influences the electrical and optical properties of deposited films. Simultaneously, characterization of the HiPIMS is presented for better understanding of the discharge behaviour during the process.

## 2. Materials and Methods

### 2.1. Materials

**Figure 1:**
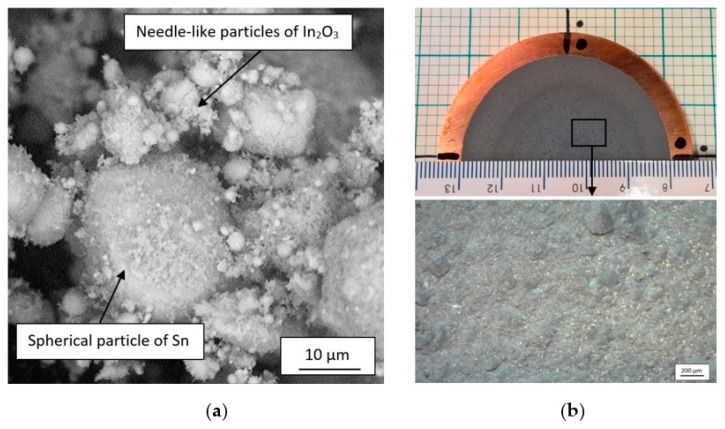
Mixture of In_2_O_3_/Sn powders (**a**) and target fabricated by LPCS with surface magnification (**b**).

The cold-sprayed targets were prepared using mixture of two commercially available powders, spherical tin (Sn) with particles size in the range of −13 + 4 µm (Libra, Trzebinia, Poland), and needle-like indium oxide (In_2_O_3_) with particles size in the range of −1.21 + 0.39 µm, forming a sponge agglomerates (VWR Chemicals, Leuven, Belgium). In order to enhance tin oxide content necessary for ITO target production, metal powder was annealed in furnace at a temperature of 220 °C for 6 h without a protective atmosphere. Tin increased oxidation starts with 150 °C [43], however higher temperature, e.g., above 200 °C, guarantees formation of SnO_2_ over SnO [44]. The In_2_O_3_ and oxidized Sn powders were mixed in the volume ratio of 3:1 using a vibrating one-ball mill (agate ball diameter of 52 mm) with vibration amplitude set to 10 mm and milling time of 30 min. Final mixture of the powders is presented in Figure 1a. A copper M1E disc with dimensions of 65 × 5 mm was used as a substrate. Prior to spraying the copper substrate was cleaned in acetone and grit-blasted with alumina (mesh 20).

### 2.2. Targets Preparation

A low-pressure cold spraying (LPCS) unit—DYMET 413 (Obninsk Center for Powder Spraying, Obninsk, Russia) was applied to spray the targets. Untypical circular de Laval nozzle with the length of 250 mm and outlet diameter of 8 mm was chosen to increase tin deposition efficiency. Further oxidation of tin powder was possible by using preheated air as the working gas. A manipulator holder with attached spraying gun moved according to the designed helix path with pitch of 4 mm. Targets were fabricated with following spraying process parameters: (i) working gas pressure *p* = 0.6 MPa, (ii) working gas temperature *T* = 300 °C, (iii) linear speed *V* = 10 mm/s, (iv) powder feed rate $\dot{m}$ = 50 g/min, and (v) spray distance *l* = 20 mm. To increase thickness of the targets, three coating layers were deposited. A self-prepared target is presented in Figure 1b.

### 2.3. Magnetron Sputtering Process

Magnetron sputtering deposition was performed using WMK-50 planar magnetron source, which is capable of operation with continuous power density up to 50 W/cm^2^. LPCS hybrid-type targets were successfully applied without a visible erosion zone. The magnetic field induction over the race-track region (component parallel to the target surface) was about 85 mT. To avoid arcs formation only the cathode assembly was used. Thin films were deposited in various mixtures of Ar:O_2_ (further sample notation in the text is given after dash): (i) 100:0 vol.%—100Ar, (ii) 90:10 vol.%—90Ar, (iii) 75:25 vol.%—75Ar, (iv) 50:50 vol.%—50Ar, and (v) 0:100 vol.%—0Ar. Oxygen rich atmosphere was applied to oxidize tin atoms. The sputtering process was performed with a total pressure of working gas and the vacuum chamber of 0.8 Pa and 0.001 Pa, respectively. Substrates were fixed 80 mm from the surface of the magnetron cathode. The power supply consisted mainly of a tank capacitor, high voltage supply and two high power switches. The tank capacitor was charged by the high voltage supply and consecutively discharged by the magnetron cathode. The repetition frequency of the charge and discharge steps was set to 0.3 Hz. A low duty cycle of HiPIMS pulses enabled deposition at low substrate temperature. The films were deposited within 300 pulses, with a total time of 900 s and voltage of 900 V. The number of the pulses was controlled by AVR Arduino Uno microcontroller electronics. The HiPIMS setup was described in detail in our previous work [45].

HiPIMS process started with formation of LC resonant circuit. Two alternate pulses with sinusoidal shape were generated, e.g., sputtering and inverse pulse, having duration of 20 µs and 25 µs, and peak current of 1600 A and 500 A, respectively. The combination of parameters unbalanced the magnetron. Nevertheless, a high value of the discharge peak current temporarily unbalanced it even further. As a result, the conditions of electrons avalanche were temporarily degraded. It is assumed the magnetron source used in the experiments was extremely unbalanced and can be classified as the device of 6th group in accordance with the Gencoa Ltd. classification.

### 2.4. Coatings and Thin Films Characterization Methods

The microscope analysis of powders, LPCS coatings and ITO films were performed using Nikon Eclipse MA 200 optical microscope (OM) (Minato, Japan) and Tescan VEGA 3 SBH SEM microscope equipped with SE, BSE detectors, and EDS system for elemental analysis. Small samples were cut from the LPCS coatings, mounted in epoxy resin and polished to prepare metallographic specimen. Thickness of the coatings was measured in the thickest and thinnest point of the cross section of three various specimens and a mean range value was determined. EDS linear analysis was performed with accelerating voltage of 15 kV and magnification of 5000×.

Structural properties of as-deposited ITO thin films were determined using X-ray diffraction in grazing incidence mode (GIXRD) employing PANalytical Empyrean diffractometer (Malvern Panalytical, Malvern, UK) with PIXel3D detector and Cu Kα X-ray source with the wavelength of 1.5406 Å. Analysis of the crystallite size was performed according to the Scherrer’s equation [46]. Optical properties were assessed based on transmission characteristics, which were measured using coupled Ocean Optics QE 6500 and NIR256-2.1 spectrophotometers (Orlando, FL, USA) and coupled deuterium-halogen Micropack DH-2000-BAL light source in the 300–2200 nm wavelength range.

Focused ion beam (FIB) (Gallium) was used to cut out specimens from specific regions of ITO film for further studies on SEM microscope. The thickness of deposited films was measured in the centre of the sample using FEI Helios NanoLab 600i (Thermo Fisher Scientific, Waltham, MA, USA). ImageJ (ver. 1.50i) software was used to analyse porosity of LPCS coatings and tin micro-particles inclusions in ITO films. ImageJ calculates area and pixel value statistics of image selections defined by the user based on threshold intensity. The semi-quantitative graphic analysis was made on five SEM images of the samples with magnification of 1000.

**Figure 2:**
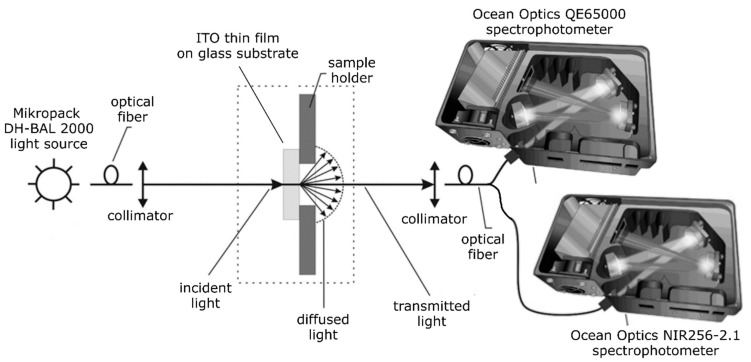
A scheme of experimental setup for the transmission spectra measurements.

Electrical properties of the deposited ITO thin films were determined using Jandel Multiheight Four Point Probe Stand (Jandel Engineering Limited, Leighton Buzzard, UK) and Keithley 2611 System SourceMeter. Optical properties were assessed based on transmission characteristics, which were measured using two spectrophotometers, i.e., Ocean Optics QE 6500 for a 200–1000 nm wavelength range and NIR256-2.1 for a 900–2200 nm wavelength range. Moreover, a Micropack DH-2000 BAL light source was used, which was consisted of coupled deuterium and halogen lamps. The fundamental absorption edge of investigated samples was determined by glass substrate parameters and was equal to about 318 nm. Optical fibers were used to illuminate ITO thin films and to direct the light beam transmitted through the film to the spectrophotometers. The experimental setup for measuring the transmission spectra is shown in Figure 2.

## 3. Results

### 3.1. Cold-Sprayed Targets

**Figure 3:**
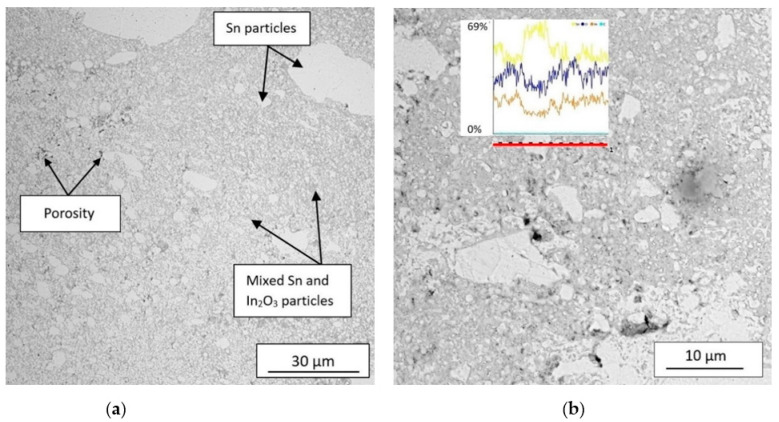
SEM micrographs of In_2_O_3_/Sn coating (**a**) and linear elemental analysis of selected region marked with red line (**b**). Annotation of the diagram lines: yellow—tin, blue—oxygen, orange—indium, azure—carbon. The particles were identify by EDS.

The microstructure of In_2_O_3_/Sn coatings deposited by LPCS is shown in Figure 3. It is clearly visible that coatings are dense with low micro-porosity of 1.36%. The thickness of the coatings was in the range of 720–1080 μm and resulted from surface waviness typical for cold-sprayed coatings. Coatings showed uniform distribution of indium oxide and tin particles. It is worth stressing that the morphology of powder particles strongly influences the structure uniformity and mechanical properties of a polycrystalline composite [47]. However, locally bigger tin particles, e.g., 20–30 µm in diameter, are noticeable (see Figure 3a). Wang et al. [48] concluded that super-fine ceramic particles can be easily rejected from the surface of metal particles during impact leaving an unreinforced region. A linear EDS analysis proved similar tin and oxide content in mixed In_2_O_3_/Sn particles regions with a little lower content of indium (see diagram on Figure 3b). The peak period of the yellow line on the diagram (Figure 3b) indicate location of tin particle. LPCS is dedicated for composite metal–ceramic mixtures deposition [49]. Nevertheless, the optimum size of metallic particles for efficient deposition ranges from 5 to 50 μm and depends strongly on the powder material [50]. Therefore, deposited coatings obtained homogeneous distribution of components, despite very small ceramic particles. In respect of further application of the coating as a target, the homogeneity was mandatory. Moreover, it was required to oxidize tin particles in hybrid-type composite coating prior to magnetron sputtering of oxide films. Therefore, air with a relatively high temperature of 300 °C was used as a working gas. Temperature of the gas stream at the end of the nozzle measured with thermocouple was 196 °C. Despite supersonic flow with high velocity, tin oxidized in the gas stream due to relatively small size particles [51]. It is well known that oxidation of metal powder increases critical velocity of single particles [52,53]. However, tin shows low value of both critical and erosion velocity in comparison to other metals [39]. Consequently, deposition efficiency of oxidized tin was not decreased.

### 3.2. HiPIMS Deposition Process and ITO Films

A self-designed HiPIMS power supply powered the magnetron source with voltage pulses of 900 V. To ensure conditions for repeatable ignition of sputtering discharge the gas pressure was increased from typical for WMK-50 source value of 0.25 Pa to 0.8 Pa. The anode ring was not used to eliminate the uncontrolled arcs formations caused by the high value of magnetron source current (1600 A). The magnetron source was encircled by the dielectric pipe and covered with a glass cap to reduce the space for discharge plasma. The sputtering sub-pulses generated high density plasma and consequently increased the ionization rate of sputtered atoms. The deposition rate of ITO films was in the range of 2.0–2.7 nm/min and increased with decreasing admixture of oxygen. According to results presented by Rezek et al. [42], HiPIMS method ensures definitely higher deposition efficiency compared to other magnetron sputtering techniques. 

**Figure 4:**
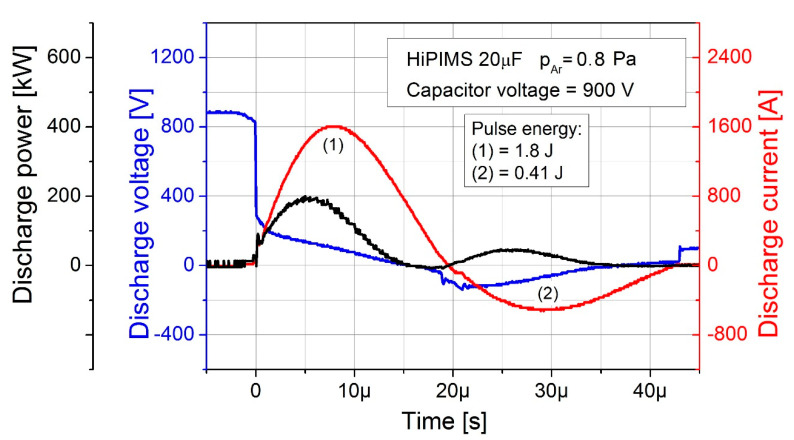
Diagram of discharge current, voltage and power vs. time for a single deposition pulse at capacitor voltage of 900 V.

Figure 4 presents variation of the discharge current and voltage versus time. A discharge step of the tank capacitor was determined as a single deposition pulse with sinusoidal characteristic (see red line in Figure 4). The pulse consisted of two sub-pulses: (i) the sputtering sub-pulse delivering the energy of 1.8 J (marked as 1) and (ii) the reverse sub-pulse delivering the energy of 0.4 J (marked as 2). Therefore, the total delivered discharge energy was equal to 2.2 J. The average discharge power, calculated as the number of deposition pulses multiplied by the energy of such pulse and that divided by the deposition time, was about 0.73 W. The sub-pulses referred to the negative or positive potential at the target, respectively. The discharge power curve took the form of a wave and arose from multiplying discharge voltage by discharge current (see black line in Figure 4). The value of peak discharge current in the sputtering sub-pulse was up to 1600 A and thus unbalanced the magnetron source. As a result, temporary reduction in the magnetic field by the electron drift current occurred. Consequently, the effectiveness of gas ionization by electrons was temporarily reduced as well. This phenomenon can eliminate formation of metallic micro-particle inclusions in deposited film [45].

**Figure 5:**
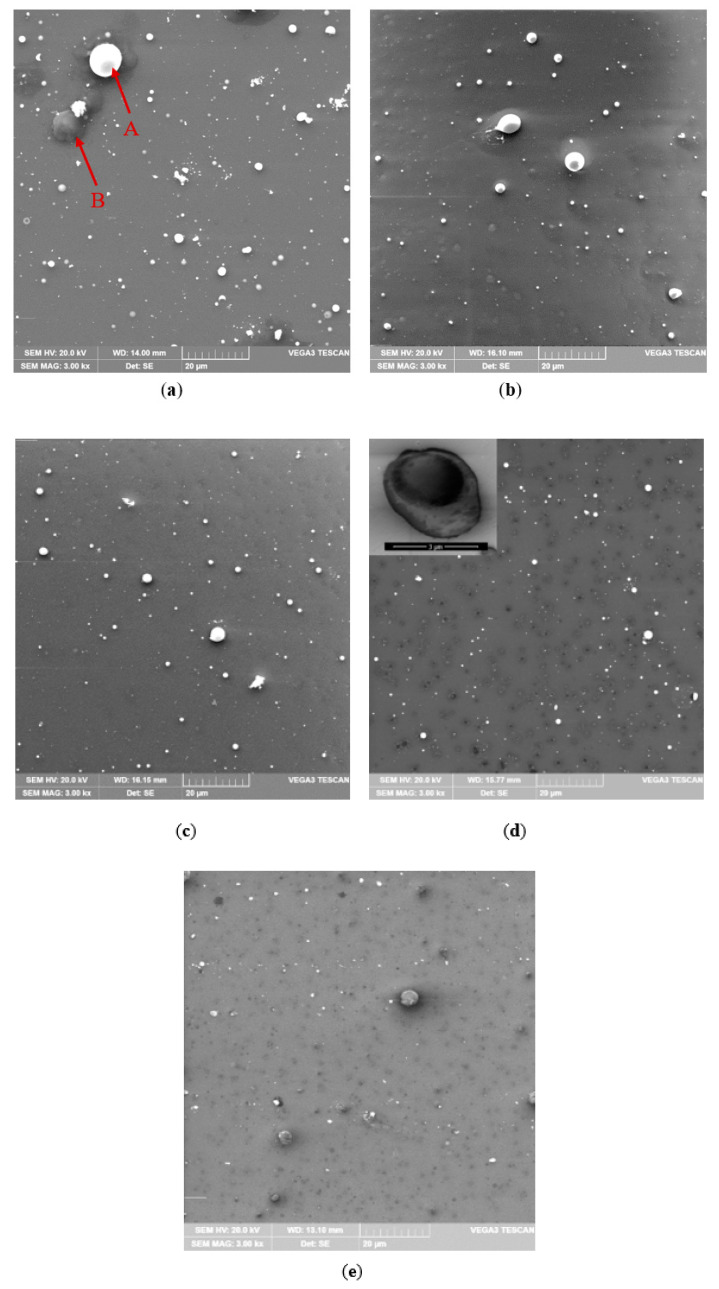
SEM (SE) micrograph of tin microparticles inclusions on ITO film surface deposited with mixture of 100Ar (**a**), 90Ar (**b**), 75Ar (**c**), 50Ar (**d**) and 0Ar (**e**). A—tin particle, B—In_2_O_3_ inclusion. The particles were identified by EDS.

**Figure 6:**
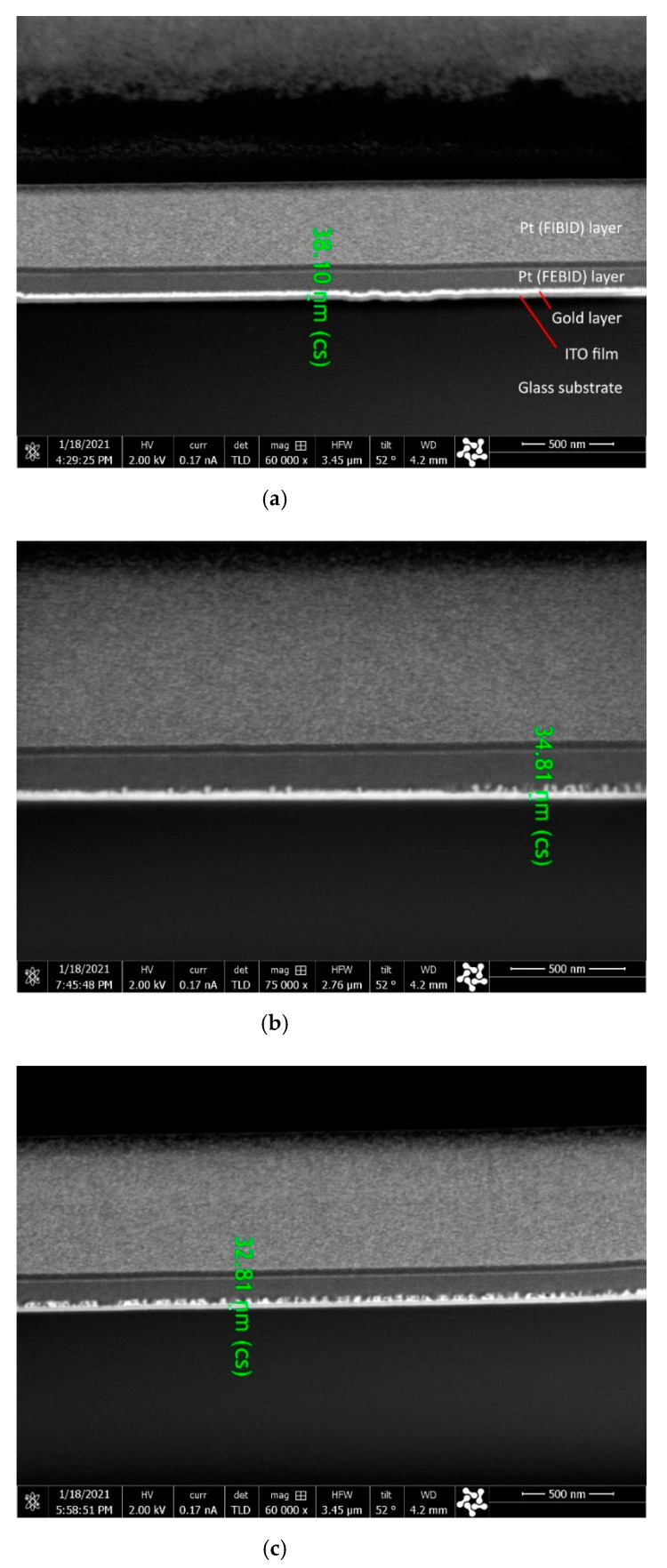
Cross-section SEM micrograph of 100Ar (**a**), 50Ar (**b**) and 0Ar (**c**) ITO film.

All deposited films showed crack free surface (Figure 5) and structure (Figure 6) as well. Thicknesses of ITO films were in the range of 32.8–38.1 nm and thus it influenced insignificantly the transparency. The major factor affecting the films thickness was the working gas atmosphere responsible for tin oxidation. Therefore, the thickest film was deposited in the atmosphere of pure argon (sample 100Ar), while the thinnest with pure oxygen (sample 100Ar) (Figure 6). In the case of ultra-thin films (thickness below 50 nm), the oxygen content in gas atmosphere influences not only film thickness [42], but refractive index of the film as well [54].

**Figure 7:**
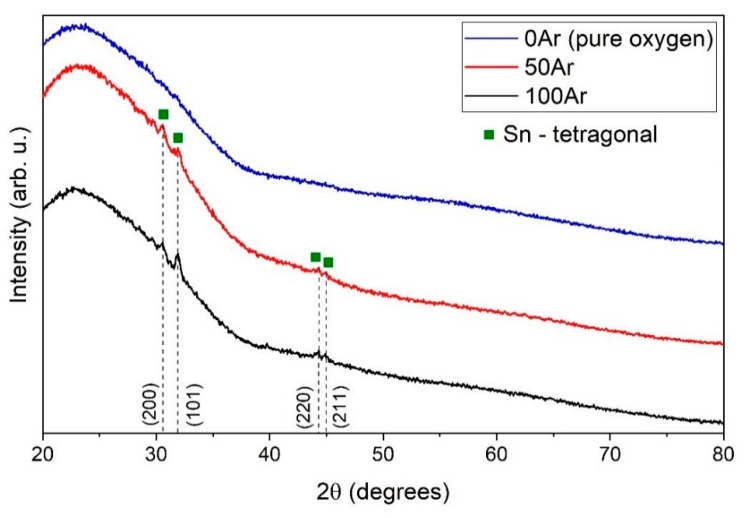
GIXRD diffraction patterns obtained for sputtered ITO films.

**Figure 8:**
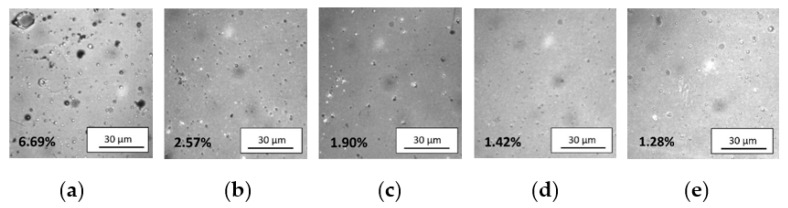
Surface morphology (OM) and percentage quantity of tin microparticles in ITO films deposited with the use of Ar:O_2_ mixture: 100Ar (**a**), 90Ar (**b**), 75Ar (**c**), 50Ar (**d**), and (**e**) 0Ar.

The GIXRD patterns of all samples are characterized only by patterns of amorphous ITO (Figure 7). Formation of amorphous ITO structure resulted from combining low-temperature magnetron sputtering process and ultra-thin thickness of the films. It should be emphasized that the initial stage of ITO growth on glass substrate normally begins with amorphous structure [55,56]. Nevertheless, in this study thermal energy necessary for crystallization was dissipated by short pulse durations [57]. ITO thin films undergo an amorphous—polycrystalline phase transition at ~200 °C [58], which increases optical and electrical properties [59]. On the other hand, amorphous structure can be desirable due to solving ITO etching residue problems in large-size 3D display devices [60]. What is more, amorphous structure is typical for ITO film deposited by low-temperature sputtering process on temperature sensitive substrates, e.g., polymers [61]. Further GIXRD analysis showed presence of metallic tin nanocrystallites with the size of 20–22 nm and a tetragonal structure [62] in ITO films deposited with pure argon (100Ar) and in the Ar:O_2_ mixture containing 50% Ar (50Ar) (Figure 7). This result can be coupled with large inclusions having diameter up to 15 µm, which were revealed on the 100Ar film surface (Figure 5a). It is assumed that tin microparticles visible in the SEM (Figure 5) images are in fact agglomerates of tin nanoparticles. It arises from unbonded metallic tin in composite target, which sputtered without oxidizing atmosphere causing pure metallic tin inclusions. Moreover, pure argon generated the highest discharge currents and thus increased possibility of microparticle inclusions. Even a short time of the sputtering sub-pulse was not able to block micro-particles ejection. Nevertheless, addition of oxygen to argon resulted in significant reduction in inclusions. The oxygen gas reacted with tin and decreased microparticles diameter below 3 µm (Figure 5b–e). Furthermore, increasing oxygen content decreased discharge current value. Eventually, tin microparticles quantity decreased from 6.69% in 100Ar sample to 1.28% in 0Ar sample (see Figure 8).

**Figure 9:**
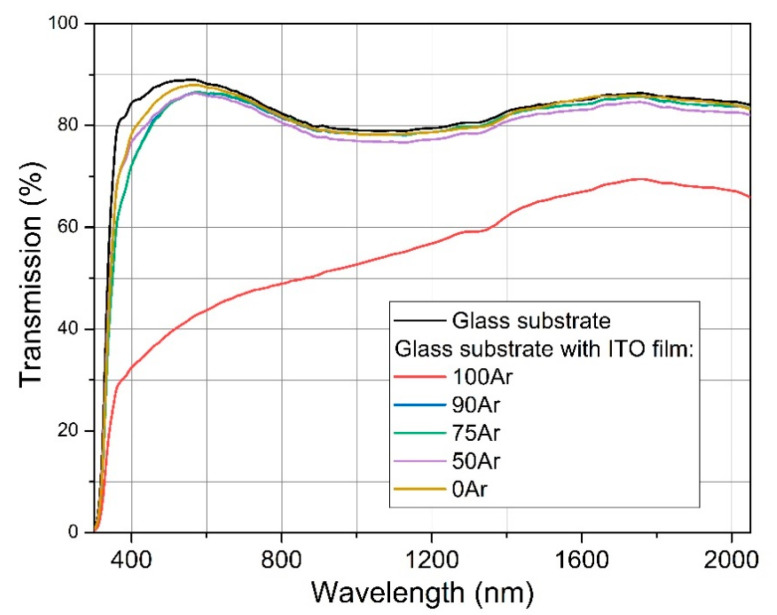
Light transmission measurements of ITO films.

Figure 9 shows results of light transmission measurements of bare glass substrate and substrates coated with ITO thin films deposited in various Ar:O_2_ mixtures. The average transparency in the visible wavelength range is quite similar for samples deposited with the use of Ar:O_2_ mixtures of 90Ar, 75Ar, 50Ar and in pure oxygen. Moreover, deposited thin ITO films had only a slight influence on the transparency of bare glass substrate. For example, the transmission coefficient at λ = 550 nm for glass substrate was equal to ca. 89%, while for sample with ITO thin film deposited in pure oxygen the transmission was about 88%. Increasing the argon content in the Ar:O_2_ mixture during magnetron sputtering up to 90% resulted only in a slight decrease in transmission coefficient to ca. 86%. However, deposition of ITO thin film in pure argon atmosphere significantly deteriorated average transparency of the final sample. For this sample, the transmission coefficient at λ = 550 nm was only about 42%. It arises from tin microparticle inclusions. Nevertheless, the addition of oxygen to argon eliminated the problem. Tin easily reacted with oxygen forming fine tin oxide particles and restrain formation of pure tin micro-droplets inclusions. Our previous results showed that applying extremally unbalanced magnetron source reduce the micro-particles inclusions [45]. On the other hand, Rezek et al. [42] noticed that sputtering efficiency from metallic target is significantly higher compared with (partly) oxidized target and thus a coverage of the target by oxide decreases with increasing the power density in HiPIMS process. Therefore, metallic targets ensure higher sputtering rate. What is more, according to Zhao et al. [54] the growth behaviour of thin films from metallic target is significantly affected by Ar:O_2_ gas and can be described by following factors: (1) the oxidation of target surface proceed more effectively with higher oxygen content, (2) the critical nucleation size depend strongly on to the partial pressure of film-forming atom, and (3) sputtering rate of the target declines in time, resulting in an expanding diffusion of adsorbed atoms and promoting formation and growth of the grains on the film surface. Consequently, a full control of process parameters, particularly the target power density and oxygen flow rate, is mandatory while applying HiPIMS process.

**Figure 10:**
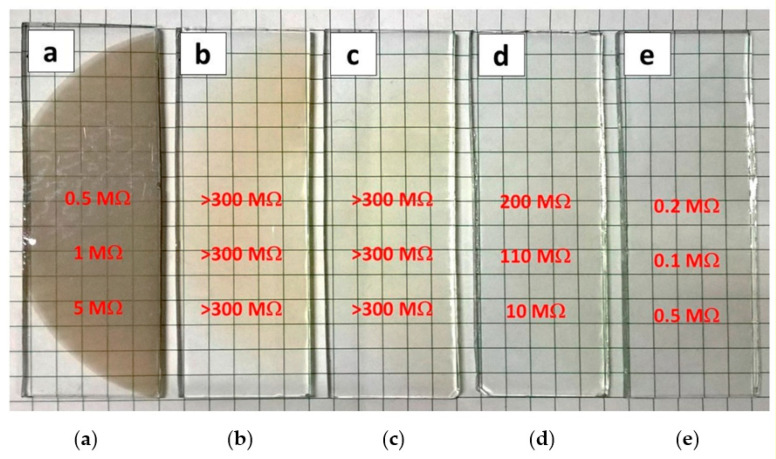
ITO films obtained after 300 deposition pulses in total time of 900 s with the use of Ar:O_2_ mixture: 100Ar (**a**), 90Ar (**b**), 75Ar (**c**), 50Ar (**d**), and 0Ar (**e**) and measured resistance.

Presence of tin micro-particles affected the resistance of the coating as well. The results of electrical measurements are presented in Figure 10. A low resistance of 0.5 MΩ (resistivity of about 2 Ω·cm and sheet resistance of 5 × 10^4^ Ω/sq) showed sample 100Ar. Moreover, the resistance increased from the centre of the film towards its borderline to 5 MΩ due to decrease in coating’s thickness (see Figure 10a). Samples 90Ar and 75Ar showed resistance higher than 300 MΩ, arising from oxidation of tin (Figure 10b,c). Nevertheless, significant improvement of the electrical properties showed sample 50Ar. Resistance of the film was 200 MΩ in the sample centre and decreased to 10 MΩ (resistivity of about 30 Ω·cm and sheet resistance of 1 × 10^6^ Ω/sq) on its borderline (see Figure 10d). Despite high transparency, 50Ar sample gain progressive electrical properties. Eventually, the lowest resistance of 0.1 MΩ (resistivity of about 0.03 Ω·cm and sheet resistance of 1 × 10^4^ Ω/sq) was obtained in the centre of 0Ar sample. It is worth stressing that electrical properties depend strongly on the thickness of deposited film which locally may differ. Therefore, appropriate process parameters can further improve the electrical properties of the films. Rezek et al. [42] observed that resistivity significantly decreases with increasing pulsed-averaged target power density up to 950 W/cm^2^ as a result of decreasing the grain size in film microstructure. In other studies, the bias voltages were frequently applied to the substrate to control the electron bombardment momentum and thus, to improve the film properties [63]. Since the negative ion energy depend strongly on the self-bias, it is stated that negative oxygen ions are one of the factors significantly influencing thin film properties [64]. Therefore, further research should be directed on increasing HiPIMS power density or lowering the DC self-bias.

## 4. Conclusions

In this study, hybrid-type composite In_2_O_3_/Sn targets fabricated by low-pressure cold spraying were applied in high power impulse magnetron sputtering (HiPIMS). The coatings showed dense structure with low porosity and uniform distribution of components. However, despite preheating of Sn powder in the air atmosphere and oxidizing parameters of the spraying process, tin particles remained in the coating and had to be oxidized during the sputtering process.

HiPIMS magnetron sputtering was performed using four various ratios of Ar:O_2_ gas atmosphere. The atmosphere of pure argon resulted in the worst transparency and satisfactory electrical properties. It arises from tin micro-particles inclusions and the highest thickness of ITO film. Increasing content of oxygen in the gas atmosphere resulted in significant increase in transparency due to reduction in metallic inclusions. Nevertheless, the electrical properties of the films decreased as well. Eventually, the application of the atmosphere of 100 vol.% of O_2_ enabled combines the best transparency with highest electrical properties. It should be noted that further research is needed to select the proper HiPIMS parameters and further decrease the resistivity of the film.

## Data Availability

The data presented in this study are openly available at [doi:10.3390/ma14051228].
